# The Pathogenesis of Ventral Idiopathic Herniation of the Spinal Cord: A Hypothesis Based on the Review of the Literature

**DOI:** 10.3389/fneur.2017.00476

**Published:** 2017-09-11

**Authors:** Ronald H. M. A. Bartels, Han Brunner, Allard Hosman, Nens van Alfen, J. André Grotenhuis

**Affiliations:** ^1^Department of Neurosurgery, Radboud University Medical Center, Nijmegen, Netherlands; ^2^Department of Human Genetics, Radboud University Medical Center, Nijmegen, Netherlands; ^3^Department of Orthopedic Surgery, Radboud University Medical Center, Nijmegen, Netherlands; ^4^Department of Neurology and Clinical Neurophysiology, Donders Center for Neuroscience, Radboud University Medical Center, Nijmegen, Netherlands

**Keywords:** embryology, spinal cord herniation, congenital, transdural appendix, review

## Abstract

Idiopathic ventral herniation of the spinal cord (SC) is not often encountered in daily practice. Its clinical prevalence, however, will increase through increasing awareness and more frequent use of MRI. A clear explanation of its pathophysiology has never been formulated. It was hypothesized that the findings during surgery might indicate the real causative mechanism. An extensive literature search was performed, using Embase, PubMed, and Google Scholar. Titles and abstracts were screened by two investigators, using strict inclusion and exclusion criteria. Reference lists of the full paper versions of each included article were checked. The following data were registered for the articles included: age, gender, level of herniation, relation to intervertebral disk, duration of symptoms, findings from surgery, and outcomes. Nine cases treated at our department were added. A total of 117 articles reporting on 259 patients were included. Including our cases, 268 patients were reviewed. Females outnumbered males (160/100). The mean age was 51.3 ± 12.0 years. In 236 patients, the duration of symptoms was reported: 55.5 ± 55.6 months. In 178 patients, the intraoperative findings for the herniated part of the SC were not mentioned. In 59 patients, a tumor-like extrusion was seen, without any alteration to the SC. Deformation of the SC itself was never observed. Biopsies of these structures were without clinical consequence. Based on the intraoperative findings reported in literature and the cases presented, acquired causes, such as trauma and erosion of the dura due to a herniated disk, were not plausible. We hypothesize that a non-functioning appendix to the SC can only develop during an early embryologic phase, in which several layers separate. We propose renaming this entity as congenital transdural appendix of the SC.

## Introduction

Idiopathic ventral herniation of the spinal cord (SC) is a rarely encountered entity in neurosurgical practice. Several reviews have been published (1–3). Some small case series have been described (1, 4, 5), but most cases have been reported as single cases (6–9). The entity is believed to be idiopathic, as a clear cause has never been identified in the cases published. However, a defect of the ventral dura has always been present. In other cases a relation is evident and, therefore, not considered as idiopathic. An example is the relation with a dural effect in combination with an intradural tumor and especially its removal (10, 11).

Many assumptions have been made about a possible acquired mechanism, with a traumatic or congenital etiology being the most frequently cited (9, 12–15). Both causes are general terms and have never been specified.

A comprehensive review of the literature was performed in the context of our own surgical experience in an effort to better understand the possible etiologies of this condition. Since we believe that specific clinical information, such as the motor grades and dermatomes involved, will not be useful in clarifying a possible cause, this information will not be collected and reviewed.

## Methods

The literature was searched, using PubMed, Embase, and Google Scholar until the end of September 2015. The search string included: {(ventral OR ventrally) AND [(herniated OR herniation) AND (spinal cord)]}. To provide a complete, exhaustive summary of current literature, the principles of the Prisma statement were followed (16). The main reason for this is to ensure clarity and reproducibility for future investigators.

Two reviewers checked the titles and abstracts. Inclusion criteria were ventral herniated SC without any language restriction. Exclusion criteria were non-ventrally herniated SC, a clear relation with an event causing the defect, such as surgery, traumatic root avulsion, and diseases known for the existence of dural defects or cysts. The first selection was based on titles and abstracts. Further selections were made after reading full texts. While assessing the full-text versions, references were checked for additional references. Finally, the articles were checked for double case reporting. When this occurred, the report with the largest series of cases was included. The quality of the studies was graded according to the guidelines proposed by the GRADE working group (17). Risk of bias was assessed according to the recommendations of Viswanathan et al. (18).

Nine cases included in the analysis are presented, two of whom are described in greater detail. These patients were identified by two authors (AG and RB), who prospectively collected data on these patients in a personal database in the hospital. The data will be complete, as these authors were the only ones who had operated on patients with SC herniation in our Neurosurgical Department.

Analyses were performed regarding mean age, gender, and duration of symptoms.

Values were represented as absolute numbers and, if appropriate, as mean with standard deviation (range). For statistical analysis, unpaired Student’s *t*-tests were used. Statistical significance was considered when *p* < 0.05.

## Results

The characteristics of the patients treated at the Neurosurgery Department of Radboud University Medical Center are represented in Table 1. Two of the patients are described in detail.

**Table 1:** Characteristics of patients treated at the Department of Neurosurgery of Radboud university medical center (1989–2015).

Case	Sex	Age	Duration symptoms (months)	Level	At disk level (Y/N)	Overall neurological outcome
1 (presented)	M	52	120	T4–T5	Y	Improved
2 (presented)	F	58	12	T8	N	Unchanged
3	M	54	40	T5/6	Y	Improved
4	F	46	32	T6/7	Y	Improved
5	F	49	56	T5/6	Y	Unchanged
6	M	56	30	T4/5	Y	Improved
7	F	39	24	T5/6	Y	Unchanged
8	F	60	76	T6/7	N	Worse
9	M	50	58	T5/6	N	Improved

### Case 1

A 52-year-old male fell 10 years before presentation from a scaffolding. Immediately after this fall, he noticed a diffuse sensibility loss in his right leg, which did not improve. Remarkably, he did not notice temperature changes or pain in that leg. Gradually, the strength in his left leg diminished. In recent years, this has clearly been progressive. In his lower left leg, he noticed paresthesias. He had a normal erectile function, but was unable to ejaculate.

**Figure 1:**
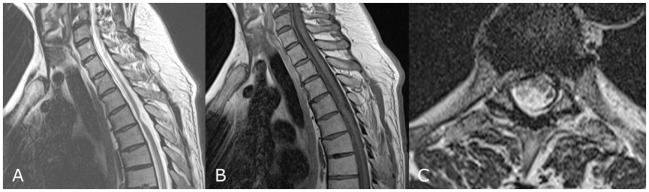
MRI of case 1 showing signs of a herniated spinal cord at T4–T5 at a sagittal T2-weighted image **(A)**, sagittal T1-weighted image **(B)**, and an axial T2-weighted image **(C)**.

On neurological examination, his left leg was slightly paretic. Vital and gnostic sensibility loss were present on the right side below T8. Within the L4 dermatoma on the left side, the vital sensibility was lost. The reflexes of both legs were pathologically increased with a Babinski’s sign on both sides. Radiological examination revealed a herniation of the SC at T4–T5 (Figure 1).

**Figure 2:**
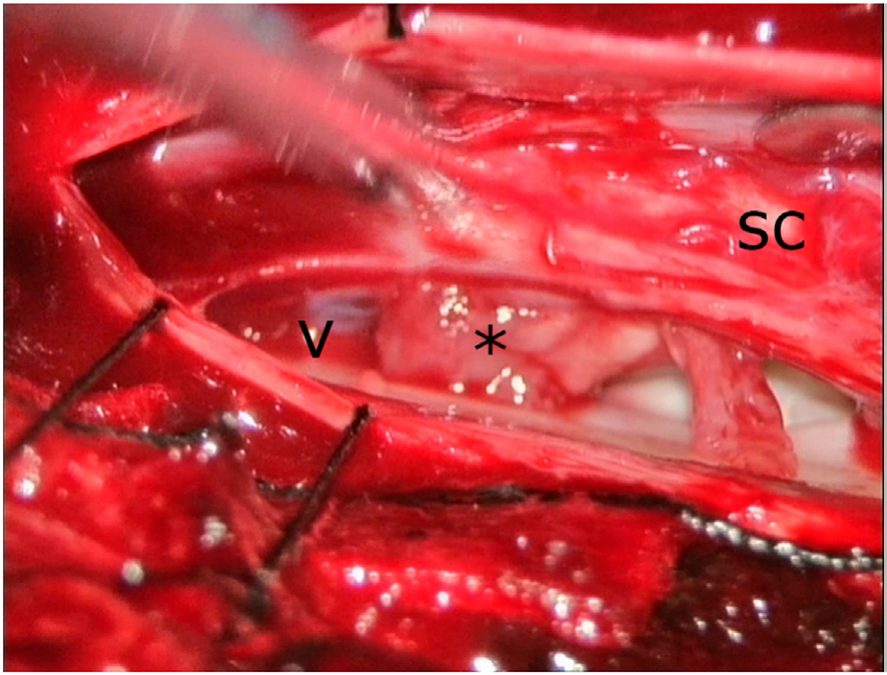
Intraoperative view from the left side. Spinal cord (SC) was slightly moved to the right side with a spatula. The edge of the dural defect (V) can clearly be seen as well as the appendix from the SC (*).

During surgery, the dura was opened after a midline approach with laminectomy T4 and T5. The dentate ligaments were transected on both sides, allowing some manipulation of the SC. On the ventral side, a defect of the dura was seen, along with many adhesions from the dura to the SC. These were sharply transected from both sides. Finally, the SC was freed from the dural defect and a mass originating from the ventral part of the SC was developed from the dural defect (Figure 2). To prevent re-herniation, an artificial graft was gently placed in front of the SC covering the defect and was fixated laterally to the dural sleeve. Throughout the entire surgery, MEPs, SSEPs, and D-waves were monitored. During manipulation, the signals diminished twice. After waiting for several minutes, the signals returned to a normal level. Post-operatively, the patient did not notice any new complaints. The neurology is his right leg remained unaltered three months after surgery, whereas the symptoms in his left leg had improved.

### Case 2

This 58-year-old female complained about temperature changes and hyperpathy in her left leg for the past 12 months. On the right side, the sensibility was different but not normal. The strength in her left leg was diminished. These complaints ascended over time, initially starting at the foot and developing further to the groin at the time of presentation.

Neurological examination revealed only a paretic quadriceps femoris muscle on the left side. Vital sensibility was disturbed on the left side below T10. The plantar reflexes on both sides were indifferent.

**Figure 3:**
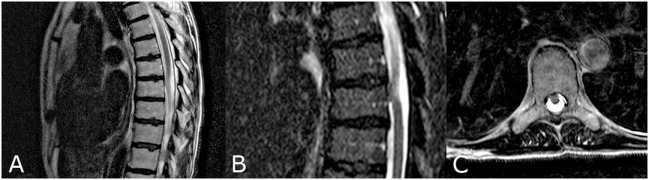
MRI of second patient disclosing a herniated spinal cord at T8 at sagittal T2 weighted image **(A)**, at a STIR weighted image **(B)**, and an axial T2 weighted Image **(C)**.

An MRI revealed a herniation of the SC at T8 (Figure 3).

**Figure 4:**
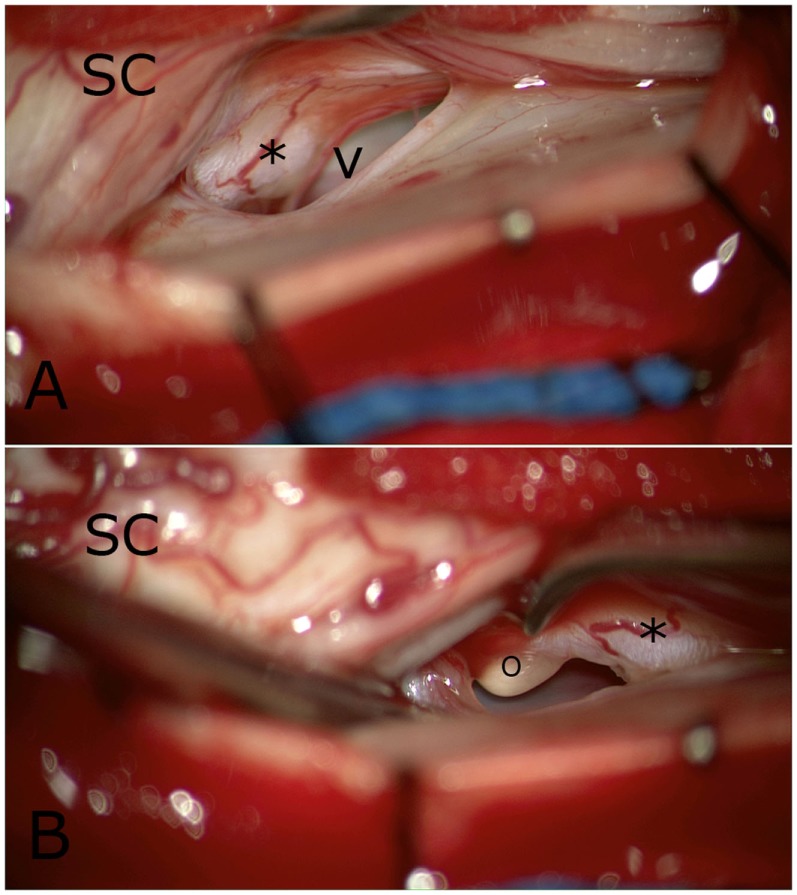
Intraoperative view from the right side. Spinal cord (SC) was disclosed and a clear tumor-like appendix was seen (*), as was the sharp edge of the ventral dural defect (V) in **(A)**. More caudally, **(B)** a yellow globule (o) was apparent that was adherent to the above-mentioned appendix.

During surgery, a laminectomy on T8 and T7 was performed. The dentate ligaments were transected. While gently mobilizing the SC from the left to the right, a large defect in the ventral dura was observed. The same maneuver at the right side revealed a fixed SC with the defect. After transection of some adhesions, a tumor-like extension of the SC was seen (Figure 4) and was finally mobilized completely intradurally. To prevent re-herniation, an artificial graft was gently placed in front of the SC covering the defect and fixated laterally to the dural sleeve. Throughout the entire surgery, MEPs, SSEPs, and D-waves were monitored. Changes were not noticed. Post-operatively, the patient did not notice any new complaints.

## Review of the Literature and all Cases

**Figure 5:**
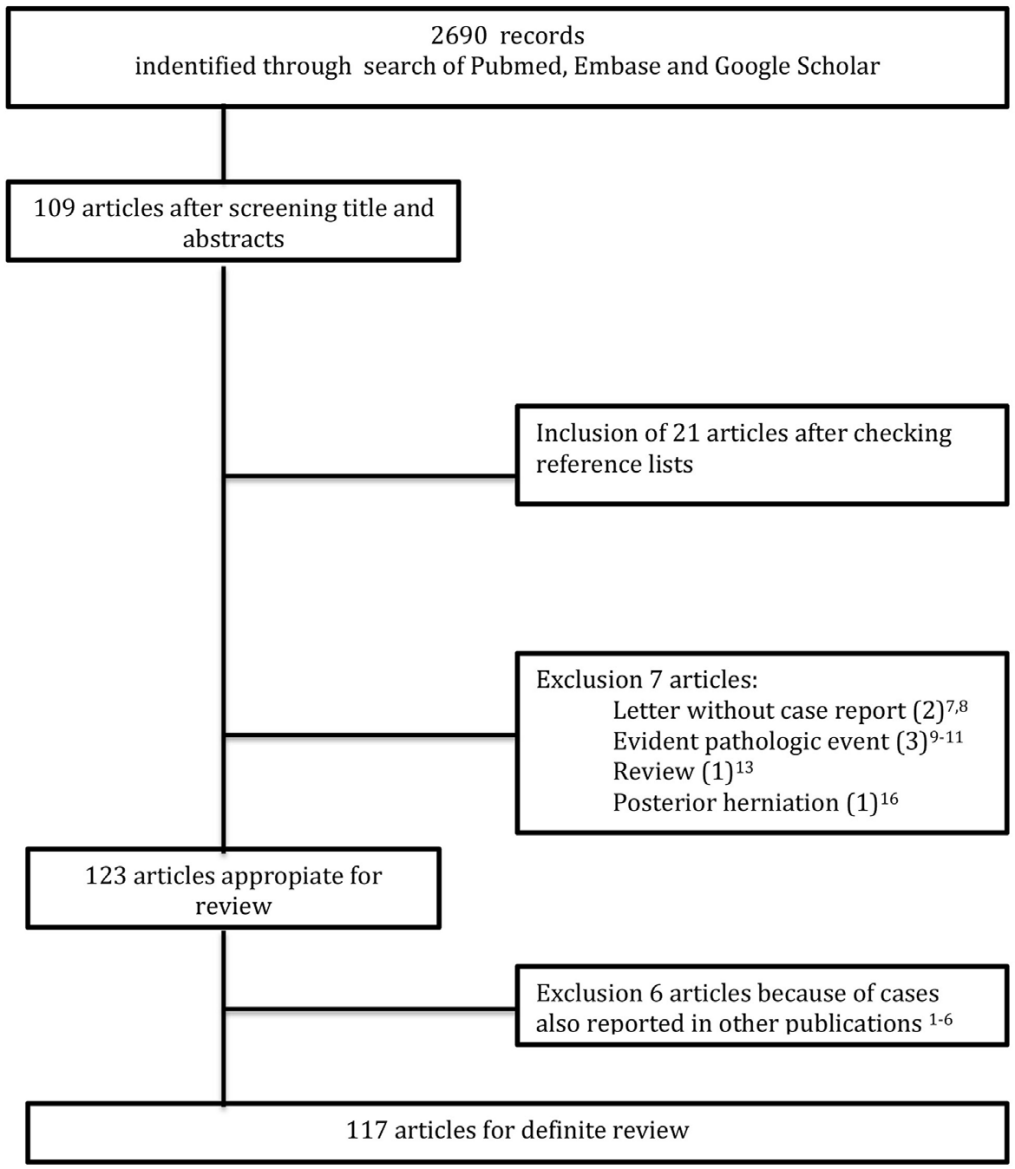
Flow of information.

The results of the literature search are shown in Figure 5. In total, 123 articles related to ventrally located herniation of the SC were selected (1–9, 12–15, 19–129). After checking for double case reporting, 117 articles were selected, reviewing 259 patients. For further analyses, we included our cases. Therefore, the total number of patients was 268.

All studies were case reports, or small retrospective case series. Therefore, the quality of the studies should be graded as low. Risk of bias was high due to the design of the studies included.

The female-to-male ratio was 164/104 (in eight cases, gender was not mentioned) or 1.6/1. The mean age was 51.3 ± 12.0 years (12–83). The mean age did not differ between men and women (*p* = 0.84). In 236 patients, the duration of symptoms was reported: 55.5 ± 55.6 months (1–432). A difference between genders did not exist in duration (*p* = 0.47).

In 135 cases, the herniation was at the disk level, whereas in 63 cases, it was not. In 70 cases, the relation to the disk was not mentioned or was radiographic investigations included. The levels of herniation of the SC are presented in Table 2.

**Table 2:** Level of herniated spinal cord (t: thoracic) with the respective frequencies.

Location	Frequency
– t1–t2	2
– t2	5
– t2–t3	18
– t3	11
– t3–t4	16
– t4	21
– t4–t5	46
– t4–t5 and t5–t6	1
– t5	7
– t5–t6	28
– t6	15
– t6–t7	31
– t7	11
– t7–t8	24
– t8	10
– t8–t9	6
– t9	1
– t9–t10	2
– t10–t11	2

Total	268

*In one case, a double herniation occurred*.

In 14 cases, the instituted treatment was not mentioned. Seventeen patients were treated conservatively. For two of them, the outcome of the final follow-up was not reported. Two of them improved (14, 129), and the other 13 cases remained unaltered.

Including our nine patients, 237 patients underwent surgery. There were no striking differences between our cases and those reported in the literature. In all cases, it was reported that the herniated portion was dissected from adhesions to the dural defect. For 178 patients, a description of the herniated part was not provided. In the remaining 59 patients, the intraoperative findings (Table 3) were reported. The SC at that level had an abnormal appearance in 18 cases, whereas all descriptions relating to the herniated part (39 cases) resembled a tumor-like entity or appendix. The outcome in the surgically treated patients was unknown in 21 (7.9%) cases, unchanged in 50 (18.7%) cases, improved in 183 (70.3%) cases, and worsened in 14 (5.2%) cases. Very few cases developed a recurrence. It was also remarkable that none of our patients experienced a CSF leakage post-operatively, although no attempt was made to close the dura. The literature makes no mention of any such occurrences either. It should be emphasized here that the follow-up was a variable. Since the effect of surgical treatment was not the purpose of this study, we did not report on patient follow-up.

**Table 3:** description of intraoperative findings and their respective frequencies in absolute numbers.

Description of intraoperative findings	Frequency
– A “tongue” of the anterolateral funiculus	1
– A small lobule of herniated spinal cord (SC) was seen, tethered by the rostral arcuate margin of the dural defect	1
– A small nipple of the cord was noted poking out whereas the rest of the SC was contained within the arachnoid membrane	1
– A tumor-like protuberance from the ventral aspect of the SC	1
– Anterolateral aspect was yellow-ochre colored	1
– Appendix from the SC	1
– Bulk of tissue	1
– Bulbous lobule	1
– Cord hernia	1
– Focal sclerosis	1
– Gliotic SC	1
– Glistening white structure epidurally	1
– Globule with yellowish-ocre like small part at the end of the herniated globule adherent to posterior longitudinal ligament	1
– Herniated and gliotic-appearing cord	1
– Herniated cord was found, showing prominent yellow discoloration	1
– Herniated lobule	7
– Herniated portion of the SC appeared yellowish and slightly hardened-like granulation tissue	1
– Herniated portion appeared gliotic	1
– Herniated portion was edematous and swollen	1
– Herniated SC had a gliotic appearance	1
– Herniated tissue. The fibers appeared edematous and reddish	1
– La moelle est manifestement remaniée (couleur jaune et ocre)	1
– Nerve root existed through defect, at histological examination fibrosed nervous tissue	1
– Nerve root in defect	1
– Pale, yellowish, swollen cord tissue	1
– Small round lesion	1
– Smooth, rubbery, yellowish white tumor-like sphere with flimsy capsule	1
– SC. Atrophic at the herniation level	12
– SC … had an exophytic edematous appearance	1
– SC protruded, resembling a “navel”	1
– SC was deformed and gliotc	1
– The herniated cord appeared “violaceous/pale” in color and was hardened	1
– The herniated portion of the thoracic cord exhibited a yellowish and edematous round-shaped projection	1
– The strangulated portion of the dura resembled a tumor	1
– Tumor-like appendix was seen and a yellowish globule was apparent that was adherent to the previous mentioned appendix	1
– Ventral bulge, where it had a gliotic, reddened appearance	1
– With a complex anterior herniation through a dural defect of gliotic tissue which was also tethered to the posterior longitudinal ligament	1
– Yellowish, tumor-like mass	1
– Yellowish and slightly softened	1
– Yellowish lobulated tumor-like herniation (photo)	1
– Yellowish tongue shaped	1
– Yellowish tongue-shaped projection	1

Total	59

## Discussion

Suggested causes for the development of a ventrally herniated SC vary from acquired to congenital (46, 69). Erosion of the dura due to a ruptured disk, or minor trauma, has been suggested as an acquired cause (9, 12–15, 113). A common factor in all the reports was that the possible mechanism was not specified. In most instances, authors cited previous authors.

In attempting to explain the etiology of ventral herniation of the SC without an obviously predisposing event, some considerations should be taken into account. First, the adult SC does not have any growing potential unless a neoplasm is involved. Second, spontaneous opening in the ventral dura cannot be expected, considering its consistency and lack of movement. If movement should play an important role, it is along a smooth connective tissue: the posterior longitudinal ligament (PLL). Third, the SC is extremely sensitive to trauma and any protrusion fixed into a dural defect will be accompanied by a severe neurological deficit. Fourth, most patients appeared with symptoms in their sixth decade and had gradually developed signs and symptoms, sometimes presenting with an acute temporary neurological deficit after a minor fall several years before the onset of the symptoms Finally, the herniated part was biopsied or even resected without any neurological deficit.

Based on these considerations, a traumatic etiology does not seem plausible. A traumatic dural tear would need a high-energy impact with a severe concomitant neurological deficit. Erosion of the dura over a herniated disk would ultimately lead to an intradurally located herniated disk with dorsal displacement of the SC. We concluded that an acquired origin seemed highly unlikely, to say the very least.

A congenital cause is very often cited without being specified (4, 7, 101, 102).

An embryologic explanation for the so-called herniation of the SC is, however, plausible. During the formation of the neural tube, neural crest cells arise at the dorsolateral aspects of the neural tube. The neural crest cells are adjacent to the neural tube and spread along it to its ventral side (130, 131). During this process, mesenchymal cells from the somites intermingle with the neural crest cells forming the meninx primitiva (130–133), a precursor of the meninges. At the gestational age of 30–32 days, the neural tube has already been covered by a single cell layer representing the future pia (130).

The layer at the ventral side of the neural tube and dorsal side of the intervertebral disk of the vertebral body, consisting of neural crest and mesenchymal cells, was reported to be thicker than laterally or dorsally (134, 135). It can be subdivided in the following three layers: (1) an outer perichondral one adjoining the vertebral body, (2) an intermediate one that will form the PLL, and (3) an internal one forming the ventral dura mater (133).

**Figure 6:**
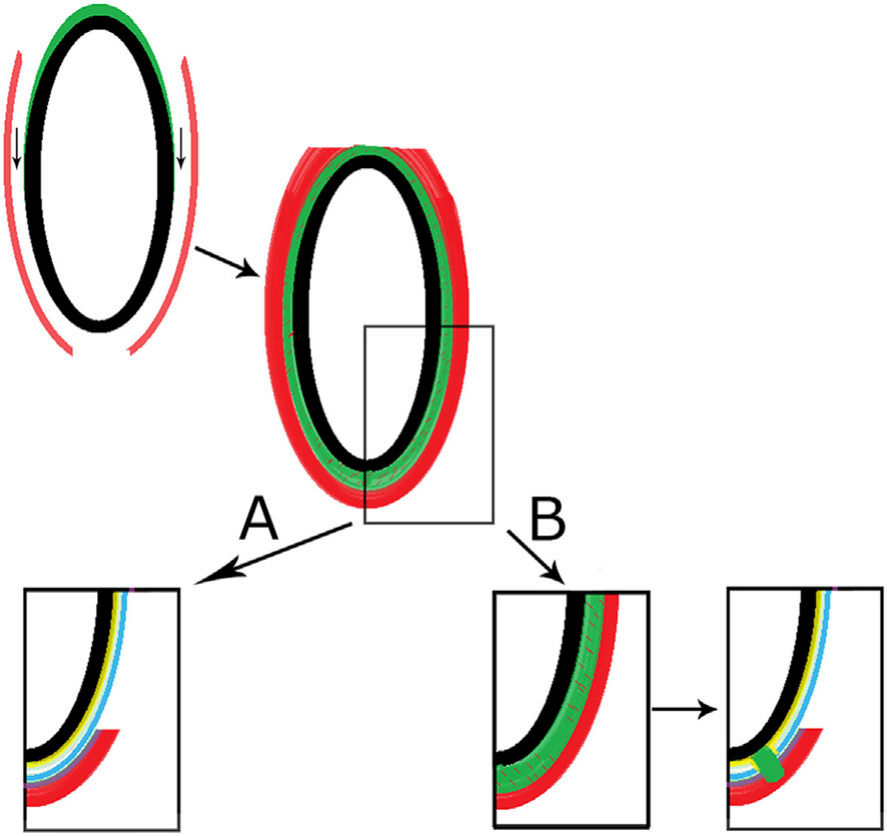
Schematic presentation of proposed hypothesis. In the upper left corner, the neural tube is shown (black) with adjacent somites (red). The neural crest (green) is formed at the dorsolateral aspects of the neural tube and migrates to the ventral aspect of the neural tube (arrows). In the middle, the neural crest cells have been intermingled with mesenchymal cells from the somites forming the meninx primitiva. **(A)** The normal embryologic development is shown with the formation of the pia mater (yellow), the dura mater (blue), the posterior longitudinal ligament (violet), and the vertebral mesenchyme (red). The formation of the congenital transdural appendix of the spinal cord is depicted in panel **(B)**. A local aggregation of neural crest cells is formed (green without red dashes) and the transdural appendix is formed, while perforating the dura, sometimes the posterior longitudinal ligament, and on rare occasions a little cavity within the vertebral body. Color legend: black: neural tube; red: somites (later vertebral mesenchyme); green (neural crest); yellow (pia mater); blue (dura mater); and violet (posterior longitudinal ligament).

It is therefore hypothesized that, within this thicker layer, neural crest cells accumulate and differentiate into neural tissue instead of dura. They have this propensity because they also form the spinal ganglia. Then, an aggregate of non-functioning neuronal cells (appendix) would be established adjoining the SC, (partly) covered by pia, and causing a defect in the dura, in the PLL (sometimes present), or on rare occasions, a small cavity within the vertebral body (Figure 6). The formation of the appendix could take place between 30 and 60 days of gestational age, depending on the specimens investigated, as the differentiation of dura mater took place before this gestational age (130, 133).

The neural tissue does not contribute to any neurological function. Biopsy of a part of the SC, or even re-section of a part will generally lead to neurological deficit. The absence reported in some cases of a neurological deficit after a biopsy had been performed proves that it was a non-functional tissue. However, some nervous cells were seen on histological examination (19, 32, 36, 43, 49, 88).

The transdural appendix will cause tethering of the SC and could become clinically relevant. A severe (temporary) neurological deficit can occur after a minor accident. Neurological deficits can also occur gradually during aging. The gradual occurrence of symptoms or their sudden emergence after a minor fall was reported in all of the cases in the literature, as well as in our own series.

The hypothesis is supported by findings described on radiological examination: nuclear trail (78), clefts in the vertebral body at the level of MRI pathology (1, 96), and a cavity in the vertebral body at the level of pathology (29). A nuclear trail or clefts indicates that the somites did not properly fuse. Cavities could only be present if these were present when the abnormality developed, since the SC fixed in a dural opening would not have eroded the bone.

This hypothesis also explains the absence of any post-operative CSF leak. Although a defect in the dura was always clearly seen and was often even enlarged to complete dissection of the protrusion, no attempt was made to close the defect. This could only happen if the dura was firmly fixed with surrounding ligament, which confirms our theory. It can also explain the atrophic appearance of the SC at the involved level. Due to the tethering of the SC, gradually circulatory changes will occur with atrophy as a consequence.

Although this hypothesis explains the findings from surgery, as well as the clinical picture, two important questions still need to be answered: (1) why did this entity only occur at the thoracic levels and (2) what was the primary cause? For example, was it genetic?

If this theory is correct, the term idiopathic ventral herniation of the SC is a misnomer. Congenital transdural appendix of the SC would be a more accurate name, since its cause is clearly defined. The term does not refer to an active process but to the result of a developmental disorder: an inert segment adherent to the SC.

This study has several limitations. We performed a review of the literature. In order to be complete and transparent, we used the guidelines proposed by Prisma. In light of that, a less informed reader could misjudge the quality of this paper as high grade evidence. This was certainly not the intention of the authors. A theory about the origin of idiopathic ventral SC herniation was formulated, based on observations and knowledge of the structure of the dura and vulnerability of the SC reported in literature. We are fully aware that solid proof was not provided, and assumptions were made. However, every observation seemed to fit into the hypothesis. Formation of a hypothesis is the first step toward initiating further investigations. We are the first to formulate this hypothesis. Therefore, we were convinced that this study is a valuable contribution to the literature.

## Conclusion

We have formulated a hypothesis to explain the development of a form of ventral tethering of the SC. This hypothesis is based on the results of our systematic review, including some of our own cases, and focused on the intraoperative findings. The etiology of this entity is assumed to be entirely embryologic. We believe that ventral SC herniation might be more appropriately referred to as congenital transdural appendix of the SC.

## Ethics Statement

The Ethics Committee CMO Arnhem-Nijmegen approved the trial. Patients cannot be identified. Since their dates were retrospectively assessed, informed consent was waived by the ethical committee.

## Author Contributions

RB and JG designed the study, collected data, analyzed the data, interpreted data for the work, drafted the work, critically revised it, and finally approved it and agreed to be accountable for all aspects of the work in ensuring that questions related to the accuracy or integrity of any part of the work are appropriately investigated and resolved. AH, HB, and NA analyzed the results, drafted and critically revised the manuscript, and finally approved it. All agreed to be accountable for all aspects of the work in ensuring that questions related to the accuracy or integrity of any part of the work are appropriately investigated and resolved.

## Conflict of Interest Statement

The authors declare that the research was conducted in the absence of any commercial or financial relationships that could be construed as a potential conflict of interest.
